# Disruption of protease A and B orthologous genes in the basidiomycetous yeast *Pseudozyma antarctica* GB-4(0) yields a stable extracellular biodegradable plastic-degrading enzyme

**DOI:** 10.1371/journal.pone.0247462

**Published:** 2021-03-17

**Authors:** Natsuki Omae, Yuka Sameshima-Yamashita, Kazunori Ushimaru, Hideaki Koike, Hiroko Kitamoto, Tomotake Morita

**Affiliations:** 1 Research Institute for Innovation in Sustainable Chemistry, National Institute of Advanced Industrial Science and Technology (AIST), Tsukuba, Japan; 2 Institute for Agro-Environmental Sciences, National Agriculture and Food Research Organization (NARO), Tsukuba, Japan; 3 Bioprocess Research Institute, National Institute of Advanced Industrial Science and Technology (AIST), Tsukuba, Japan; University of Tsukuba, JAPAN

## Abstract

The yeast *Pseudozyma antarctica* (currently designated *Moesziomyces antarcticus*) secretes a xylose-induced biodegradable plastic-degrading enzyme (PaE). To suppress degradation of PaE during production and storage, we targeted the inhibition of proteolytic enzyme activity in *P*. *antarctica*. Proteases A and B act as upper regulators in the proteolytic network of the model yeast, *Saccharomyces cerevisiae*. We searched for orthologous genes encoding proteases A and B in the genome of *P*. *antarctica* GB-4(0) based on the predicted amino acid sequences. We found two gene candidates, Pa*PRO1* and Pa*PRO2*, with conserved catalytically important domains and signal peptides indicative of vacuolar protease function. We then prepared gene-deletion mutants of strain GB-4(0), ΔPa*PRO1* and ΔPa*PRO2*, and evaluated PaE stability in culture by immunoblotting analysis. Both mutants exhibited sufficient production of PaE without degradation fragments, while the parent strain exhibited the degradation fragments. Therefore, we concluded that the protease A and B orthologous genes are related to the degradation of PaE. To produce a large quantity of PaE, we made a Pa*PRO2* deletion mutant of a PaE-overexpression strain named XG8 by introducing a PaE high-production cassette into the strain GB-4(0). The ΔPa*PRO2* mutant of XG8 was able to produce PaE without the degradation fragments during large-scale cultivation in a 3-L jar fermenter for 3 days at 30°C. After terminating the agitation, the PaE activity in the XG8 ΔPa*PRO2* mutant culture was maintained for the subsequent 48 h incubation at 25°C regardless of remaining cells, while activity in the XG8 control was reduced to 55.1%. The gene-deleted mutants will be useful for the development of industrial processes of PaE production and storage.

## Introduction

With the continual increase in the demand and consumption of plastics, plastic pollution has become an urgent environmental issue worldwide. Poly(butylene succinate-*co*-adipate) (PBSA) and poly(butylene succinate) (PBS) are biodegradable aliphatic polyesters with good elongation at break and other physical properties similar to polyethylene. They have been developed as environmentally friendly packaging materials and can reduce environmental pollution by replacing conventional commercial plastics (e.g., single-use cutlery, agricultural mulch films, compost bags, coffee capsules, and tea bags). However, the use of these biodegradable products is limited because the increased strength reduces biodegradability, and vice versa. The degradation also depends on the environment [1–3]. Therefore, the development of a rapid degradation technique is required to accelerate the commercialization of these biodegradable plastics (BP).

We have studied enzymes that promote the degradation of BP products at the end of life. We discovered yeast strains of the genera *Pseudozyma* and *Cryptococcus*, which produce enzymes that efficiently degrade aliphatic polyesters [4]. The gene encoding the BP-degrading enzyme, PaE, was cloned and sequenced from *Pseudozyma antarctica* [5]. PaE production was induced by xylose, and large-scale production of PaE was demonstrated by a xylose-fed batch culture using a jar fermenter [6]. A large amount of xylanase was also secreted in the culture medium [7]. The recombinant *P*. *antarctica* strain, in which a fragment of the PaE gene (Pa*CLE1*) was introduced under the control of the xylanase promoter (PaE gene expression cassette), secreted more PaE in the presence of xylose [8, 9]. The shapes of PBSA, PBS, and commercial BP mulch films, immersed in the diluted culture filtrate, were disrupted and disappeared [6]. PaE also degrades poly(ε-caprolactone) (PCL), amorphous poly(L-lactide) (a-PLLA) [10] and poly(butylene adipate) films [11]. Therefore, a PaE solution may be useful to promote the degradation of various BP products. However, during storage of PaE after large-scale production, degradation occurred; this could limit its application. Therefore, in the present study, we aimed to reduce the degradation of PaE and maintain its activity during cultivation and storage.

In *Saccharomyces cerevisiae*, the protease pathway is one of the most important protein degradation pathways [12]. Vacuoles contribute to cellular protein homeostasis by degrading senescent, superfluous, and damaged proteins and organelles [13, 14]. Vacuolar proteinases, including proteases A and B, are responsible for bulk protein degradation. They contribute 40% and 85%, respectively, to overall protein of *S*. *cerevisiae* degradation under vegetative and sporulation conditions [15]. These proteases also play an important role in self-maturation, as well as in the maturation of other proteases [13, 14]. Notably, they regulate the proteolytic network. Thus, we focused on these two proteinases to develop a method for stable production of PaE.

In the present study, we identified two protease gene candidates (Pa*PRO1* and Pa*PRO2*) in the genome of a typical PaE producer, *P*. *antarctica* GB-4(0). Their identification was based on catalytically important residues, motifs, and conserved domains. The two gene-deletion mutants, ΔPa*PRO1* and ΔPa*PRO2*, were capable of producing PaE without degradation fragments during a 5-day cultivation period, followed by 8 days of incubation at 25°C. The degradation fragments reappeared when using gene-complemented mutants. Hence, we concluded that the two genes, Pa*PRO1* and Pa*PRO2*, contribute to the degradation of the extracellular enzyme PaE in the strain GB-4(0). Furthermore, we achieved high and stable production of PaE by deleting Pa*PRO2* from the strain XG8, a PaE over-expression strain based on the strain GB-4(0).

## Materials and methods

### Strains and plasmids

The wild-type *P*. *antarctica* strain GB-4(0), used as the host strain, was isolated from rice husks [4] and deposited in the Genebank of the National Institute for Agrobiological Sciences, Japan (accession no. MAFF 306999). Strain XG8 was used as a host for gene disruption of Pa*PRO2*. The high-producing PaE strain XG8 was constructed from GB-4(0) by introducing a xylose-inducible PaE gene expression cassette. The PaE gene of GB-4(0), under control of the xylanase promoter, was linked to an *Escherichia coli* neomycin phosphotransferase-encoding gene (Neo^r^) under the *P*. *antarctica* strain T-34 homocitrate synthase promoter (_P_*LYS20*) [8]. All of the yeast strains were cultivated at 30°C.

### Sequence analysis

We searched for candidate orthologous genes of *PEP4* encodong the gene product Pep4p (GenBank accession: AAB63975.1) and *PRB1* encoding the gene product Prb1p (GenBank accession: AAB65027.1) against GB-4(0) draft protein sequences using the FASTA program [16] with an E-value threshold at 10^ -15. Pair-wise alignment was carried out using Clustal Omega [17], and conserved domains were analyzed by BLASTP [18] with default settings.

### Gene deletion in GB-4(0)

The two genes in strain GB-4(0), Pa*PRO1* and Pa*PRO2*, were deleted by homologous recombination. Fragments containing the nourseothricin resistance gene and coding region of Pa*PRO1* or Pa*PRO2* were prepared as gene-deletion fragments (S1 Fig) and introduced into cells of strain GB-4(0). All primer sets and information for the strains used in this study are listed in S1–S3 Tables.

Fragments for gene deletion were generated by three-step PCR. The first step was to create two gene fragments, "up" and "down," by PCR (1 cycle of 94°C for 2 min; 30 cycles of 98°C for 10 s, 62°C for 30 s, and 68°C for 1 min; and 1 cycle of 68°C for 7 min) using genomic DNA from GB-4(0) as the template. The second step was amplification of the “NAT cassette” gene fragment, harboring natMX4, using a pAG25 plasmid containing a nourseothricin resistance gene (natMX4) (provided by EUROSCARF at the Institute of Molecular Biosciences, Johann Wolfgang Goethe University, Frankfurt, Germany). The natMX4 gene was developed as a selection marker for *S*. *cerevisiae* [19]. The third step was binding the three fragments, "up," "NAT cassette," and "down", by PCR (1 cycle of 94°C for 2 min; 30 cycles of 98°C for 10 s, 62°C for 30 s, and 68°C for 3 min; and 1 cycle of 68°C for 7 min). After verification and purification, nested PCR was performed under identical PCR conditions to those used for overlap PCR. The product was then purified. In this study, all gene amplification by PCR was performed using the KOD-FX DNA polymerase (Toyobo, Osaka, Japan).

Transformation was conducted by the lithium acetate method as previously described [20], with the following minor modifications. Cells were mixed with TFB (40% PEG 3350, 0.2 M lithium acetate); after incubation at 37°C for 1 hour, fresh YPD liquid was added. The mixture was then incubated for 2 hours at 30°C with shaking at 200 rpm. Transformed cells were selected with YM plates containing 100 μg/mL of nourseothricin (Jena Bioscience, Erfurt, Germany).

The resulting colonies were isolated and screened by colony PCR to identify positive colonies harboring the introduced DNA. PCR was conducted in two steps: positive screening was used to select colonies that exhibited amplification of the gene fragment, and negative screening was used to remove colonies that retained the original gene from their genomic DNA (S1 Fig). Gene deletion was then confirmed by genome sequencing with a next-generation sequencer. Only one deletion cassette was detected in each mutant.

### Plasmid complementation of deleted genes

Gene fragments of Pa*PRO1* and Pa*PRO2*, including ~1-kb flanking regions, were PCR-amplified from the genomic DNA of strain GB-4(0) and inserted into pEmp (pUVX1-neo [8]) using *Eco*RI/*Hin*dIII or *Eco*RI/*Bam*HI sites, as a multiple copy type of gene-expression vector. The resulting plasmids were named pEmp-*PaPRO1* or pEmp-*PaPRO2*, respectively (S2 Fig). Subsequently, these two plasmids were transformed into DH5α cells, as confirmed by colony PCR. The nucleic acid sequences of fragments in the plasmids were also confirmed (FASMAC, Kanagawa, Japan). The two plasmids carrying the Pa*PRO1* or Pa*PRO2* gene, including the 1-kb flanking regions, were introduced into cells of the gene-deletion mutants, ΔPa*PRO1* and ΔPa*PRO2*, as well as the wild-type, employing the same method described for deletion mutants. Neomycin was used for colony selection instead of nourseothricin. Transformants were verified by colony PCR.

### Flask cultivation

A pre-culture was prepared overnight in yeast malt broth (YM) at 30°C and 200 rpm. The culture (300 μL) was transferred to 30 mL of 3 x FMM medium (8.0% xylose, 0.3% yeast extract, 0.2% NaNO_3_, 0.06% KH_2_PO_4_, and 0.06% MgSO_4_-7H_2_O in a 300 mL flask) and cultured for 5 days at 30°C and at 200 rpm. Cells in the culture were then removed by centrifugation. The supernatant was transferred to a sterilized flask and incubated at 25°C without shaking.

### Cultivation in a jar fermenter

Strain XG8 or ΔPa*PRO2*-XG8 was cultivated in a jar fermenter, as described previously [8]. Briefly, 18 mL of pre-culture was added to the 3-L jar fermenter containing 1.5 L of PaE production medium (0.2% yeast extract, 0.2% NaNO_3_, 0.5% (NH_4_)_2_SO_4_, 0.04% KH_2_PO_4_, 0.04% MgSO_4_·7H_2_O, and 2% xylose). After 24 h cultivation, xylose fed-batch cultivation was performed by adding feeding medium (0.3% yeast extract, 0.05% KH_2_PO_4_, 0.01% MgSO_4_·7H_2_O, and 20% xylose) at a rate of 300 mL/d. The cultivation conditions were as follows: aeration rate, 2 LPM; agitation rate, 500 rpm; temperature, 30°C; and pH, 6.0 (controlled with a 14% ammonia solution, which also served as a nitrogen source). After cultivation for 24, 48 or 72 h, 1 mL of culture was harvested and centrifuged at 20,000 × *g* for 10 min. The pellets were dried at 105°C for 6 h, and the dry cell weight was measured to evaluate cell growth. At the same time, the emulsified PBSA-degrading activity of PaE in the supernatant was measured. After 72 h cultivation, the culture was collected and used for the PaE stability tests, as described below.

### SDS-PAGE and immunoblotting analysis of PaE

To evaluate the stability of PaE in the three strains, culture supernatants of the wild-type GB-4(0), and of the ΔPa*PRO1* and ΔPa*PRO2* mutants, were centrifuged at 20, 000 × *g* for 5 min and subjected to SDS-PAGE. The band corresponding to PaE was detected by immunoblotting, as follows. Heat-denatured samples (5 μL) were electrophoresed on acrylamide gels (mini-PROTEAN TGX gels; Bio-Rad, Hercules, CA, USA) and then transferred to PVDF transfer membranes with 0.45-μm pores (Immobilon-P; Merck-Millipore, Burlington, MA, USA) using a semi-dry transfer cell (Bio-Rad). The membrane was incubated for 1 h with blocking reagent (4% skim milk in phosphate-buffered saline containing 0.1% Tween 20 (PBST)), and then overnight with a blocking reagent containing anti-PaE antibody (1:1,000) [4]. After washing the membrane three times, it was treated with enhanced chemiluminescence (ECL) peroxidase-labeled anti-rabbit antibody (GE Healthcare, Chicago, IL, USA) in PBST for 4 h. The membrane was treated with ECL Prime Western Blotting Detection Reagent (GE Healthcare) after washing three times. Chemiluminescence of the band corresponding to PaE was detected by Fluor Chem (Alpha Innotech, San Leandro, CA, USA).

The supernatants of the jar fermenter cultivation were periodically sampled (1.67 μL) and PaE stability tests were performed based on SDS-PAGE (MULTIGEL II Mini 10/20; Cosmo Bio Co., Ltd., Tokyo, Japan). Proteins were visualized by Coomassie Brilliant Blue stain using Phast Gel Blue R (GE Healthcare, Little Chalfont, UK).

### Stability of the PaE protein in culture with cells

The stability of secreted PaE protein in cell cultures (XG8 and ΔPa*PRO2*-XG8) was analyzed as follows. After 72 h cultivation, 1 mL of each culture was transferred to seven 1.5 mL protein-low-bind-tubes and incubated at 25°C without shaking. The tubes were collected periodically (0, 4, 8, 12, 24, 36, 48 h). The collected tubes were mixed 4–5 times by inversion and centrifuged at 20,000 × g for 10 min. The supernatants were stored at -30°C until evaluation for BP degradation. PaE activity of the supernatant was analyzed. The pellets were dried at 105°C for 6 h and weighed to analyze the cell lytic tendency. PaE activity was evaluated according to a previous report [9]. All values are averages and standard deviations from twice jar-fermenter cultivation (n = 3 each).

The influence of cells on stability of secreted PaE protein in the cultures (XG8 and ΔPa*PRO2*-XG8) was analyzed as follows. After 72 h cultivation, 700 mL of each culture was collected and incubated at 25°C for 24 h (the extended 24 h incubation). After the extended 24 h incubation, each culture was centrifuged at 11,800 × g for 10 min, and then the supernatants obtained were filtrated with a 0.45 μm pore size membrane filter (Stericup, Merck-Millipore, Burlington, MA, USA) to remove cells. Each 18 mL of filtrate was transferred in 20 mL glass bottles and incubated at 25°C for 14 days. One mL of filtrates were collected periodically (0, 1, 3, 5, 7, 11, 14 days). The filtrates were stored at -30°C until evaluation for BP degradation activity. The filtrates without the extended 24 h incubation were used as the control. All values are averages and standard deviations from twice jar-fermenter cultivation (n = 3 each).

## Results and discussion

### Selection of candidate genes encoding proteases A and B in *P*. *antarctica* GB-4(0)

In *S*. *cerevisiae*, the genes *PEP4* and *PRB1* encode vacuolar proteases A and B, respectively [13]. Their main function is protein degradation, but they also play a role in the maturation and activation of other proteins [14, 21, 22]. Therefore, to suppress the degradation of PaE by deletion of the two proteinases in strain GB-4(0), we searched for orthologous genes of proteases A and B in the draft genome of this strain using amino acid sequences (*PEP4* and *PRB1*) as queries in FASTA format (Table 1, S3 Fig) [16]. Four statistically significant genes were predicted as orthologous genes of *PEP4* (3 candidates) and *PRB1* (1 candidate). Of the three *PEP4* candidates, the gene with the lowest E-value (Table 1) was regarded as the *PEP4* orthologous gene in this study. The expression frequency of the *PEP4* orthologous gene was the highest among three candidates based on the read number obtained by sequence analysis of mRNA (S4 Fig). In addition, the *PEP4* orthologous gene was the closest to *PEP4* in a phylogenetic analysis using the amino acid sequences obtained from public database (S5 Fig). Consequently, the genes were named Pa*PRO1* (accession # LC565013) and Pa*PRO2* (accession # LC565014), respectively.

**Table 1:** Predicted orthologous genes of PEP4 and PRB1 in GB-4(0). The candidate orthologous genes of proteases A and B were searched in the draft genome of strain GB-4(0) using the FASTA program and the amino acid sequences of Pep4 and Prb1 of *S*. *cerevisiae*, respectively.

Query	Query length (amino acids)	Candidate	E-value	Length (amino acids)	Aligned length (amino acids)	Identity (%)	Similarity (%)
Pep4 (Protease A)	405	#1 (PaPRO1p)	3.80E-91	561	415	56.1	75.7
		#2	5.80E-30	609	325	32.9	61.2
		#3	7.60E-23	510	335	32.2	63
Prb1 (Protease B)	635	#1 (PaPRO2p)	3.70E-61	539	444	48.0	71.6

### Sequence analysis of PaPRO1p and PaPRO2p in *P*. *antarctica* GB-4(0)

**Fig 1:**
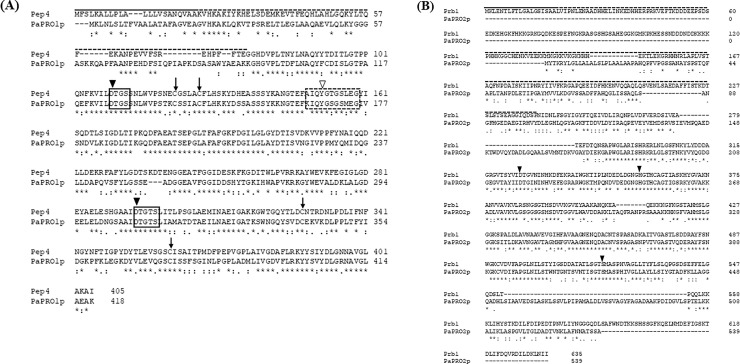
Conserved catalytic motifs in PaPRO1p and PaPRO2p. Conserved catalytic motifs were analyzed by pair-wise alignment with Clustal Omega: (A) Pep4 and PaPRO1p and (B) Prb1 and PaPRO2p.

Both of the proteases, a monomeric aspartyl endopeptidase of the A1 family of aspartic proteases encoded by *PEP4* and a serine endopeptidase of the S8 family of peptidases encoded by *PRB1*, have catalytically important residues and motifs [13]. To determine whether the gene products of Pa*PRO1* and Pa*PRO2* exhibit proteolytic activity, we used pair-wise alignment to analyze the conservation of the typical catalytic residues and domains of proteases A and B, respectively (Fig 1A and 1B).

PaPRO1p exhibited conserved functional domains, including catalytically important motifs and residues involved in building disulfide bonds identical to those in Pep4. A pre-peptide, which encoded a signal peptide directing prepro-Pep4 to the endoplasmic reticulum, was predicted in amino acids 1–22 of PaPRO1p. These corresponded to amino acids 1–22 of Pep4. We found a pro-peptide containing a vacuolar sorting signal, which is recognized by the vacuolar protein sorting receptor that targets pro-Pep4 to the vacuole. It is cleaved autocatalytically in the vacuole or transport vesicles, resulting in a mature Pep4. The pro-peptide was found in amino acids 23–92 of PaPRO1p, which corresponded to amino acids 23–76 of Pep4 [23, 24]. The catalytic motif and catalytically active aspartic acid (Asp) and flap region were highly conserved in PaPRO1p. These constitute conserved residues in eukaryotic aspartic proteinases that are presumably involved in the capture and cleavage of substrates [24, 25]. Although there is low protein sequence identity among the aspartic protease family, the three-dimensional structure is highly conserved according to a root mean square analysis of the crystal structure [25]. Protease A consists of two topologically similar lobes, each containing one catalytically active residue. The lobes are arranged symmetrically in the tertiary structure, and this folding is sustained by 268 intramolecular hydrogen bonds and two disulfide bonds [25, 26]. In the alignment, we observed that cysteines, which are used to build disulfide bonds, were conserved in PaPRO1p.

Furthermore, functional domains of Prb1 from *S*. *cerevisiae* were conserved in PaPRO2p, whereas the similarities of N and C-terminal sequences were low among them. The middle region of PaPRO2p (around amino acids 191–435) was similar to the middle region of Prb1 (around amino acids 288–534, containing three predicted catalytically active residues: D325, H357, and S519) [27].

Thus, this is the first report of two candidate genes encoding proteases A and B in a *P*. *antarctica* strain, identified based on pair-wise alignment using the amino acid sequences of Pep4 and Prb1 from *S*. *cerevisiae*.

Solid lines, pre-peptide; dotted lines, pro-peptide; solid square, proteolytic catalytic motif; closed inverted triangle, catalytically active residue; dotted square, flap region; tyrosine (Y) in the open inverted triangle, a residue conserved in aspartic proteases in eukaryote; vertical arrow, cysteine for disulfide bond. “*”, “:”, and “.”indicate identical residues, residues with strong similarities, and residues with weak similarities, respectively.

### Functional domains of PaPRO1p and PaPRO2p in *P*. *antarctica* GB-4(0)

We further evaluated the possibility of signal peptides (pre-peptides) at the N-termini of PaPRO1p and PaPRO2p by computational prediction with Phobius [28]. The results indicated that PaPRO1p and PaPRO2p have N-terminal signal peptides similar to those in Pep4 and Prb1, respectively (S6 Fig), which are experimentally validated signal peptides.

Conserved functional regions, including catalytically important residues and motifs, were estimated by homologous structure searches with BLASTP [18] (S7 Fig). PaPRO1p exhibited a conserved Fungal_Proteinase_A domain (cd05488) and was a member of the same aspartic proteinase superfamily as Pep4. The other two candidates described in Table 1 were also the member of aspartic proteinase superfamily, but conserved Pepsin like domain not Fungal_Proteinase_A domain (S8 Fig). PaPRO2p also had the conserved domains PepS8_PCSK9_ProK_like (cd04077) and Inhibitor_I9 (pfam05922), which are conserved in S8 family proteases, such as Prb1.

Thus, both gene products exhibited conserved N-terminal signal peptides and functional domains corresponding to those of vacuolar proteases in *S*. *cerevisiae*, based on the computational analyses.

### Suppression of PaE degradation products by deletion of Pa*PRO1* and Pa*PRO2*

**Fig 2:**
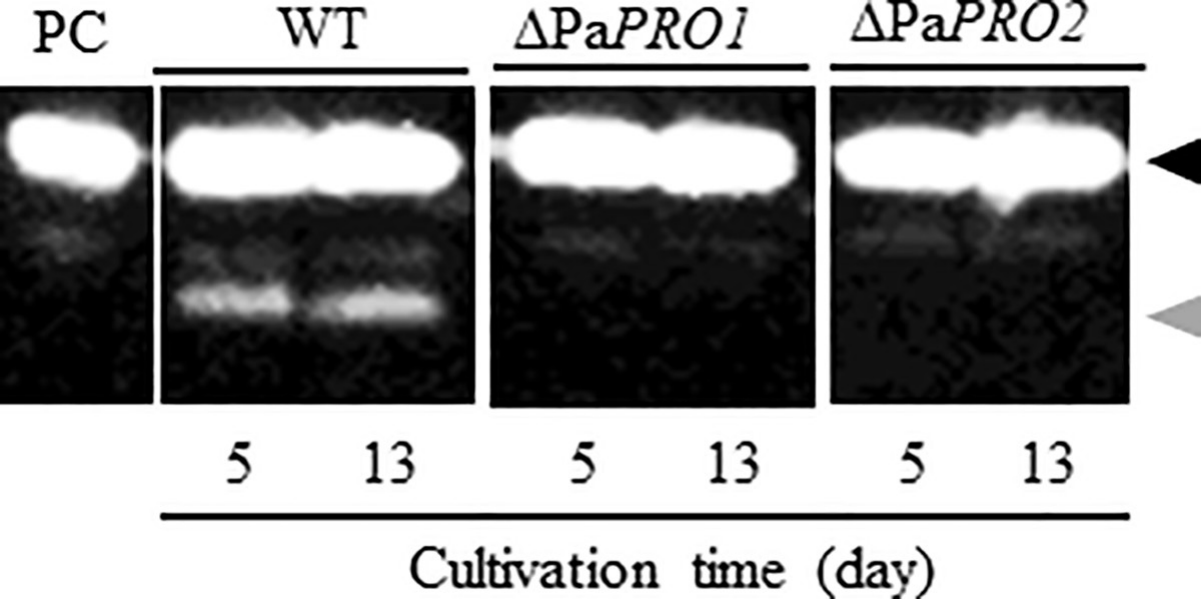
PaE production in ΔPa*PRO1* and ΔPa*PRO2* mutants. Wild-type and ΔPa*PRO1* and ΔPa*PRO2* gene-deletion mutants of strain GB-4(0) were cultured for 5 and 13 days. PaE and its degraded fragments in the supernatant were detected with anti-PaE antibody. PC, purified PaE as the positive control; black triangle, full-length PaE; gray triangles, degraded fragments of PaE in the strain GB-4(0).

To investigate the contribution of the predicted vacuolar protease genes to the formation of PaE degradation products, Pa*PRO1* and Pa*PRO2* gene-deletion mutants—ΔPa*PRO1* and ΔPa*PRO2*, respectively–were prepared in the strain GB-4(0). We then cultured these gene-deletion mutants in flasks, and the supernatants were collected by centrifugation after 5 and 13 days of cultivation. The supernatants were then subjected to immunoblotting analysis with anti-PaE antibody (Fig 2). In the supernatants of both ΔPa*PRO1* and ΔPa*PRO2*, no PaE degradation fragments were observed, whereas the fragments were detected in the supernatant of the wild-type strain. Similar results were obtained with three independent colonies derived from transformation. In addition, no decrease of the total protease activity based on activity stainig by SDS-PAGE with gelatin as a substrate was found in the both mutans (S9 Fig). By the other assay method with acid-denatured hemoglobin from bovine blood as a substrate, intracellular protease activity was significantly decreased in ΔPa*PRO1*, although the extracellular protease activity was maintained in both ΔPa*PRO1* and ΔPa*PRO2* (S10 Fig). This result suggests that ΔPa*PRO1* is probably the main regulator of intracellular proteolysis in the yeast strain. Consequently, these results strongly indicated that the predicted genes of proteases A and B, Pa*PRO1* and Pa*PRO2*, affect the generation of the degraded fragments of PaE.

### Complementation of *PaPRO1* and *PaPRO2* deletion mutants

**Fig 3:**
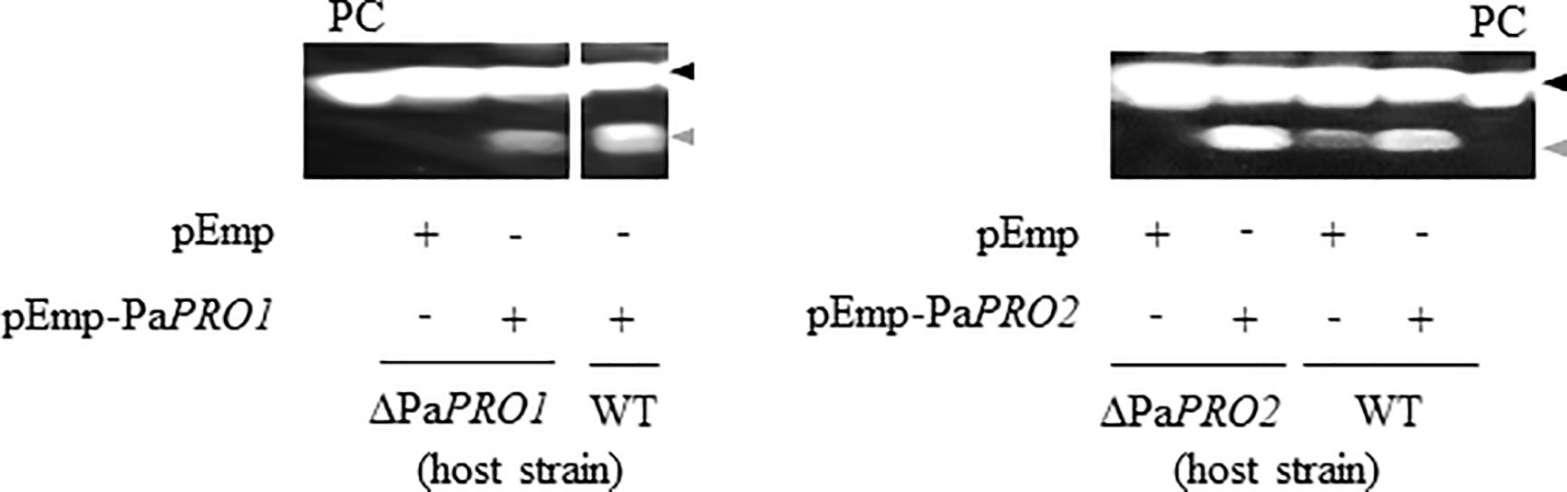
Complementation of gene deletion in ΔPa*PRO1* and ΔPa*PRO2* mutants. Secreted PaE in mutant strains (ΔPa*PRO1* or ΔPa*PRO2*) bearing the plasmids pEmp-Pa*PRO1*, pEmp-Pa*PRO2* or pEmp, and cultured for 8 days, were detected by immunoblotting with anti-PaE antibody. PC, purified PaE as a positive control; black triangle, full-length PaE; gray triangles, degraded fragment of PaE in strain GB-4(0).

We further characterized the effects of the gene products on PaE degradation by means of gene complementation with the plasmid carrying each gene, including 1-kb flanking regions (S2 Fig). The ΔPa*PRO1* mutant was transformed with plasmid pEmp as a control and pEmp-Pa*PRO1* for complementation. This resulted in strains ΔPa*PRO1*::pEmp and ΔPa*PRO1*::pEmp-Pa*PRO1*, respectively. Likewise, the ΔPa*PRO2* mutant was transformed with plasmid pEmp as a control and pEmp-Pa*PRO2* for complementation. This resulted in strains ΔPa*PRO2*::pEmp and ΔPa*PRO2*::pEmp-Pa*PRO2*. The wild-type strain was also transformed, resulting in strains WT::pEmp, WT::pEmp-Pa*PRO1*, and WT::pEmp-Pa*PRO2*. These plasmid-bearing strains were cultured for 8 days and their supernatants were then subjected to immunoblotting. PaE degradation fragments were observed in the supernatants of ΔPa*PRO1*::pEmp-Pa*PRO1* and ΔPa*PRO2*::pEmp-Pa*PRO2*. Additionally, the signal strength of degraded products from both WT::pEmp-Pa*PRO1* and WT::pEmp-Pa*PRO2* was stronger than that from WT::pEmp (Fig 3). Therefore, the introduction of Pa*PRO1* and Pa*PRO2* enhanced the formation of degraded products of PaE. Accordingly, we concluded that these gene products were responsible for the degradation of the extracellular enzyme PaE in strain GB-4(0).

### Stability of secreted PaE during fed-batch cultivation of the ΔPa*PRO2* strain that introduced a PaE high-expression cassette

**Fig 4:**
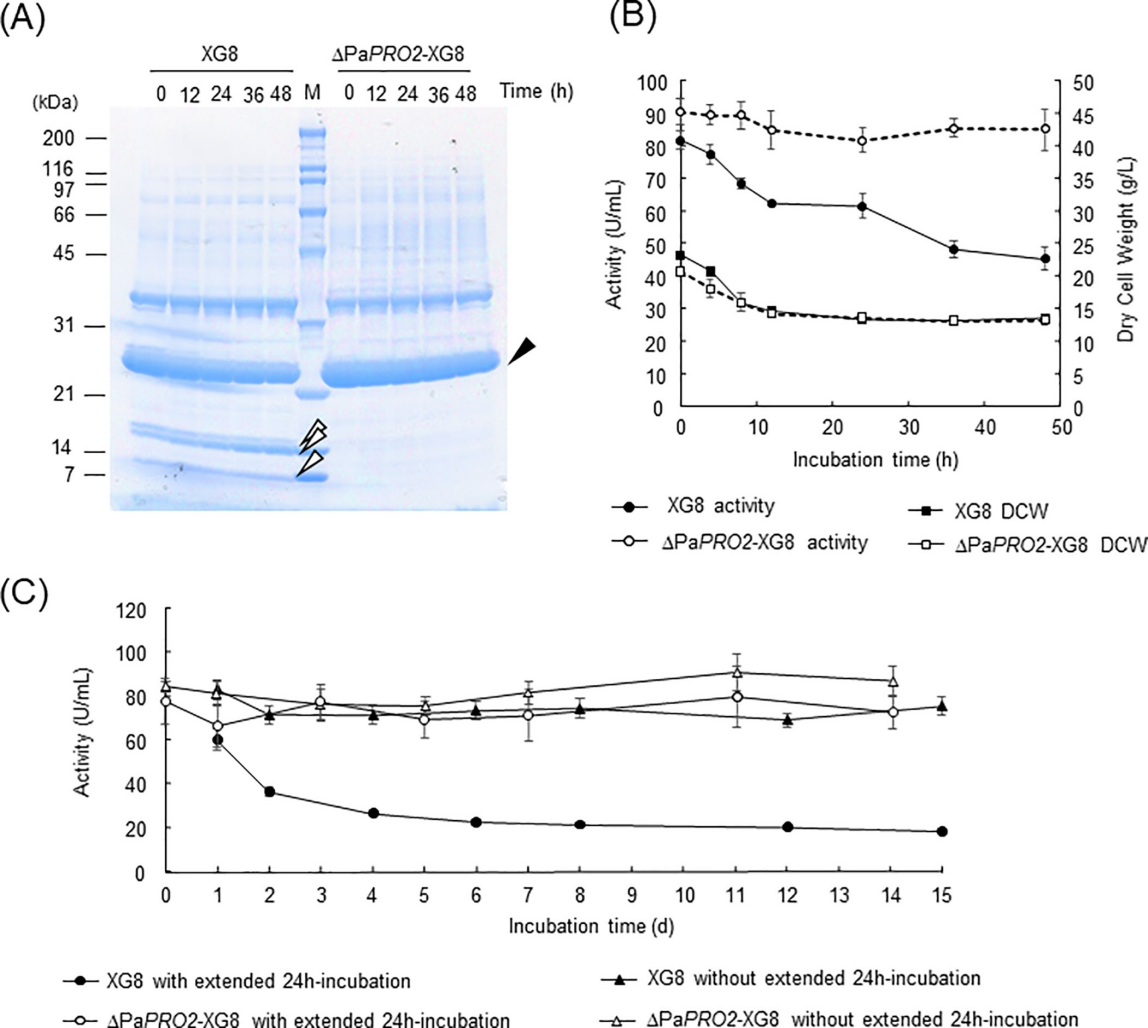
Effect of ΔPa*PRO2* on degradation and PaE activity during large-scale culture with XG8. (A) Mutant strain ΔPa*PRO2* and its parent strain, XG8, were cultured in a jar fermenter. Extracellularly secreted PaE was analyzed by Coomassie Brilliant Blue stain. Black closed triangle, full-length PaE; open triangles, degradation fragments of PaE in strain GB-4(0). (B) Dry cell weight and activity (U/mL) of PaE in strains XG8 and ΔPa*PRO2*-XG8 based on the result of 6 measurements. The standard deviation was indicated by error bar. (C) The effect of filtration removing the cells from culture on PaE activity (U/mL) in strains XG8 and ΔPa*PRO2*-XG8 based on the result of 6 measurements. The standard deviation was indicated by error bar.

ΔPa*PRO2* was used to subsequent study in the large-scale production, because ΔPa*PRO1* showed a small amount of the degraded product in the supernatant after cultivation 3L jar fermenter as preliminary experiment. To further investigate the effect of ΔPa*PRO2* on large-scale PaE production, we disrupted Pa*PRO2* in a recombinant strain, XG8, which introduced a PaE high-expression cassette in strain GB-4(0). Xylose fed-batch cultivation was carried out according to a previous report [8]. We then evaluated the PaE stability at 25°C for 48 h after cultivation. Strains XG8 and ΔPa*PRO2*-XG8 both displayed large amounts of PaE in the supernatants on SDS-PAGE. The band corresponding to the intact PaE from strain XG8 was likely to decrease compared to that of ΔPa*PRO2*-XG8 during 48 h-incubation. During the culture of strain XG8, degradation bands of PaE were observed from the start of the post-incubation. As the band of PaE became thinner, the band of degradation products appeared to become thicker. In contrast, no degraded fragment of PaE was detected in the supernatant of the culture of ΔPa*PRO2*-XG8 (Fig 4A). Additionaly, the number of thin bands at the upper part of the gel in ΔPa*PRO2*-XG8 increased compared to the strain XG8. This result suggests that there are other proteins besides PaE that avoid degradation by proteases in ΔPa*PRO2*-XG8. Although the decrease of protease activity was not detected in ΔPa*PRO2* by the two method, PaPRO2p is likely to contribute to activation of extracellular proteins including PaE and so on. Further study of proteases would allow us to understanding the proteolytic mechanism in the basidiomycetous yeast strain.

PaE activity in the supernatant of the culture of XG8 and ΔPa*PRO2*-XG8 was then analyzed during the 48-h incubation (Fig 4B) as previously described [9]. Compared to the PaE activity at the start of the post-incubation, after 48 h-incubation, the PaE activity of the culture supernatant of strain XG8 decreased to 55.1%. In contrast, that of ΔPa*PRO2*-XG8 maintained 94.0% of the activity at the start of post-incubation. The dry cell weight of strains XG8 and ΔPa*PRO2*-XG8 decreased to 60% after 48 h incubation.

Furthermore to estimate the influence of remaining cells in the culture on PaE activity, the cells were removed from supernatant of the culture by filtration. By treatment with the extended 24 h incubation after agitating cultivation, the PaE activity of strain XG8 was decreased and that of ΔPa*PRO2*-XG8 was maintained during incubation for 14 days like the above result. On the other hand, the PaE activity in the filtrates of strain XG8 stabilized without the extended 24 h incubation (Fig 4C). This result suggests that the remaining cells after cultivation causes instability of PaE, and the complete cell removal will allow to stabilize the PaE activity. However, it is hard to rapidly remove the cells from a large amount of culture in the industrial process compared with in laboratory. Therefore, to avoid the instability during removal process, the gene-deleted strain is more useful to produce the enzyme in industry. Compared with ascomycetus yeasts, many of the basidiomycetous yeasts including the genus *Pseudozyma* have been relatively under-studied in their importance for biotechnology, agriculture and food, and environmental processes [29]. The present results will contribute to provide new aspect for the industrial use of basidiomycetous yeasts.

## Conclusion

To the best of our knowledge, this is the first study to identify orthologous genes of vacuolar proteases A and B—Pa*PRO1* and Pa*PRO2*, respectively—in the basidiomycetous yeast *P*. *antarctica* GB-4(0), based on the predicted amino acid sequences. Both Pa*PRO1* and Pa*PRO2* gene-deletion mutants were able to produce the extracellular enzyme PaE without degraded fragments, while the wild-type strain produced PaE with degraded fragments. Furthermore, ΔPa*PRO2* of strain XG8 produced sufficient PaE without degraded fragments in the jar fermenter. Consequently, the use of protease-deficient mutants can contribute to the development of large-scale production and storage of PaE for industrial use.

## Supporting information

S1 Table Sequences of the primers used. (TIF)

S2 Table Primer sets to construct each strain. (TIF)

S3 Table List of plasmids and strains. (TIF)

S1 Fig Constructing deletion mutants of Pa*PRO1* and Pa*PRO2*. Disruptive fragments for Pa*PRO1* and Pa*PRO2* are shown in (A) and (B), respectively. Numbers and arrows indicate primers. Descriptions of primers and amplicons are provided in S1–S3 Tables. Deletion of the Pa*PRO1* (C) or Pa*PRO2* (D) gene was verified by PCR during positive and negative screening.
(TIF)

S2 Fig Plasmids expressing Pa*PRO1* and Pa*PRO2* used in complement experiments. Pa*PRO1* or Pa*PRO2* containing flanking sequences was inserted into pEmp plasmid using restriction sites.
(TIF)

S3 Fig Alignments of amino acid sequences of three candidates with Pep4. (A) candidate #1, (B) candidate #2, and (C) candidate #3. “*”, “:”, and “.”indicate identical residues, residues with strong similarities, and residues with weak similarities, respectively.
(TIF)

S4 Fig Expression levels of the three candidate genes of *PEP4*. The expression frequency of the three orthologous gene was analyzed based on the read number obtained by sequence analysis of mRNA using MiSeq. Total RNA was isolated from cells using ISOGEN (Wako) according to the manufacturer’s instructions as follows. Cells were ground in liquid nitrogen to a fine powder, and approximately 3 g was mixed with 15 ml ISOGEN solution, followed by the addition of 3 ml chloroform. The mixture was left at room temperature for 3 min and centrifuged at 2,300 g for 20 min. The supernatant was transferred to a fresh tube and mixed with 7.5 ml isopropanol. The mixture was left at room temperature for 10 min and centrifuged at 2,300 g for 20 min. The pellet was dried and dissolved in 300 μl of RNase-free water. mRNA was purified from approximately 200 μg total RNA using an OligotexTM-dT30<Super> mRNA Purification kit (Takara, Shiga, Japan) according to the manufacturer’s instructions. The library was prepared with TruSeq Stranded mRNA Library Prep (Illumina Inc., San Diego, CA, USA), and obtained the sequences with MiSeq (Illumina) according to the manufacturer’s instructions. Read number of each nucleotid was viewed with IGV [30].
(TIF)

S5 Fig Phylogenetic tree of aspartic proteases of *Saccharomyces cerevisiae* and *Candida albicans*, a *PEP4* ortholog of *Ustilago maydis*, and the three candidate genes for *PEP4* of *Pseudozyma antarctica* was predicted neighbor-joining method [31]. The evolutionary distance was calculated with Poisson correction method [32] and the tree was shown in scale. This analysis was performed in MEGA X [33, 34].
(TIF)

S6 Fig Predicted signal peptides of PaPRO1p and PaPRO2p. Proposed signal peptides of PaPRO1p and PaPRO2p according to Phobius are shown in (A) and (B), respectively. Horizontal axes show the distances of amino acid residues from the start codon; vertical axes show the probability of a signal peptide (maximum value = 1).
(TIF)

S7 Fig Predicted conserved domains of Pep4, Prb1, PaPRO1p, and PaPRO2p. Conserved domains were predicted by BLASTP. Numbers indicate the positions of amino acid residues.
(TIF)

S8 Fig Predicted conserved domains of candidate#1, #2, and #3. (TIF)

S9 Fig Evaluation of protease activity in culture supernatant of ΔPa*PRO1*, ΔPa*PRO2*, and wild-type by SDS-PAGE with gelatin as a substrate. Culture supernatants of ΔPa*PRO1*, ΔPa*PRO2*, and wild-type GB-4(0) were centrifuged at 20, 000 × *g* for 5 min, and subjected to SDS-PAGE containing 0.1% gelatin without heat denaturation. After electrophoresis, to remove SDS [35], the polyacrylamide gel was incubated with 2.5% tritonX-100 at 25°C for 1h, washed with distilled water, and then incubated with buffer fluids, i.e, 0.1M sodium acetate (pH5.2), 0.1M Tris-HCl (pH7.2), or 0.1M Tris-HCl (pH9.8). The gel was stained with Symply Blue Safe Stain (Invitrogen, CA). Protease activity was visualized as white band caused by gelatin degradation.
(TIF)

S10 Fig Intracellular protease activity of ΔPa*PRO1*, ΔPa*PRO2*, and wild-type. The activity of extra- (A) and intracellular (B) acid proteases was determined in the same manner as published studies [14, 36] with minor modifications. All strains were cultivated at 30°C for 5 days as with the flask cultivation in the Materials and methods section. The 1 mL of cultures after the cultivation were collected by a centrifuge at 14,000 *g* for 2 min and the supernatants were used as extracellular enzyme samples. The cell pellets were washed by 1 mL of 150 mM NaCl, resuspended in 1 mL of pure water. The resuspended cells (1 mL) were disrupted using the bead beater homogenizer (μT-12, TAITEC, Saitama, Japan) and zirconia beads (20 beads with 2 mm diameter and 3 beads with 3 mm diameter in 2 mL tube). After the disruption (3200 rpm for 20 sec and cooling on ice for 1 min, the cycle was repeated for 15 times), the disrupted cells were centrifuged at 22,000 *g* for 5 min at 4°C, then the supernatants were used as intracellular enzyme samples. The acid-denatured hemoglobin from bovine blood (Product No. H2625, Sigma Aldrich, St. Louis, MO, USA) was used as a substrate. The denaturation was performed by the incubation of hemoglobin in HCl (pH 1.8) at 35°C for 1 h, followed by pH adjustment at 3.2 by NaOH (Final concentration of hemoglobin was 20 g/L). The enzyme samples (400 μL) was mixed with 400 μL of acid-denatured hemoglobin (20 g/L, pH 3.2) and 400 μL of glycine-HCl buffer (100 mmol/L, pH 3.2) at 37°C. A portion (390 uL) of the mixture was taken at 0, 30, and 60 min, then, mixed with 700 μL of ice-cold trichloroacetic acid (TCA, 50 g/L) to stop the reaction. The TCA containing samples were incubated for 20 min at room temperature to progress the denaturation. Obtained samples were centrifuged at *22*,*000* g for 5 min at 4°C to remove denatured hemoglobin and cell-derived proteins, and these supernatants (500 μL) were mixed with 500 μL of 1 M NaOH. Finally, tyrosine-containing peptide in soluble fraction was determined using Folin & Ciocalteu’s phenol reagent (Product No. F9252, Sigma Aldrich, St. Louis, MO, USA, diluted by the same volume of pure water before the use). The NaOH added samples (1000 μL) was mixed with diluted Folin & Ciocalteu’s reagent (200 μL), incubated for 30 min at room temperature, and measured their light absorbance at 750 nm. The concentration of tyrosine-containing peptide was calculated from the calibration curve using tyrosine. One unit of activity (U) was defined as the amount of enzyme that released 1 μg of tyrosine-containing peptides per minute at 37°C.
(TIF)

S1 Raw image (TIF)

S2 Raw image (TIF)

S3 Raw image (TIF)
