# Supplementary figures and images for: Disruption of protease A and B orthologous genes in the basidiomycetous yeast Pseudozyma antarctica GB-4(0) yields a stable extracellular biodegradable plastic-degrading enzyme
Source: PLoS One. 2021 Mar 17;16(3):e0247462. doi: 10.1371/journal.pone.0247462 (PMC7968665; doi:10.1371/journal.pone.0247462)

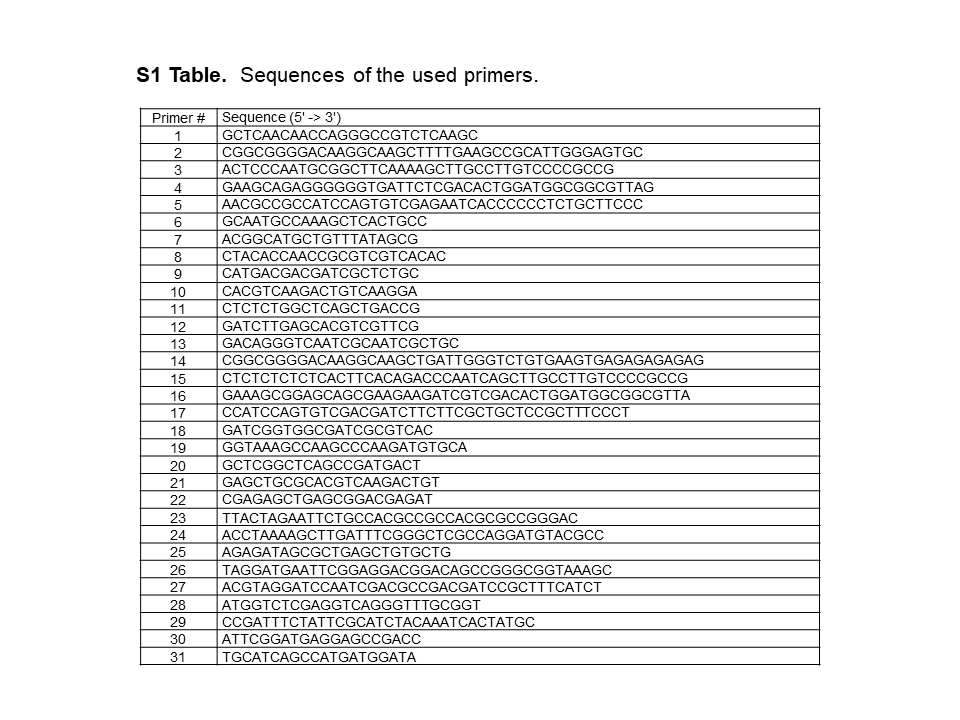

Supplement: S1 Table — (TIF) [file pone.0247462.s001.TIF]

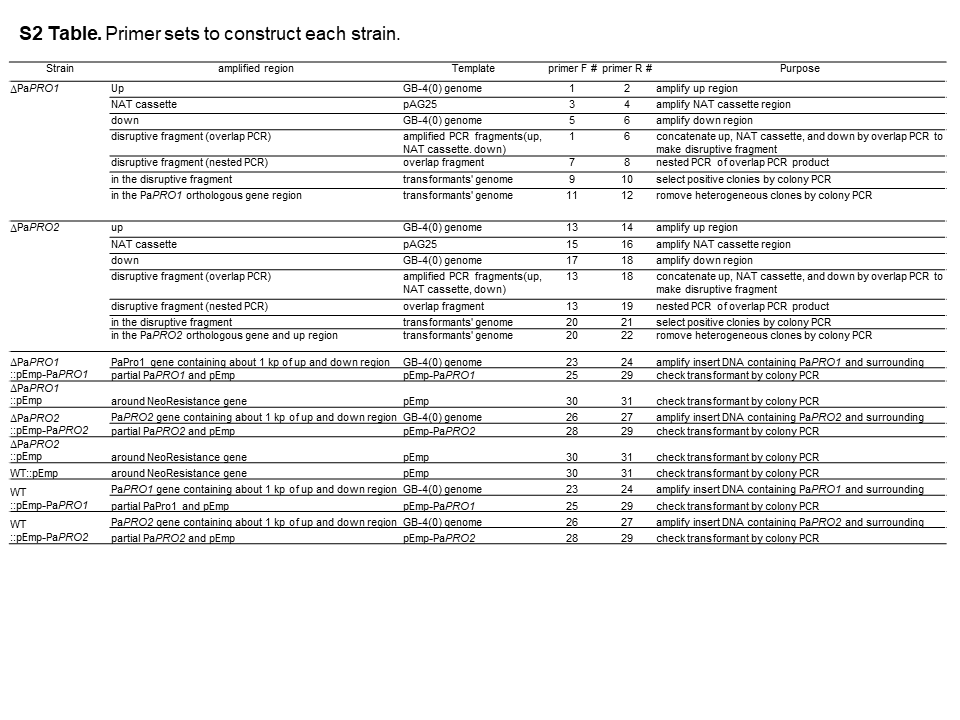

Supplement: S2 Table — (TIF) [file pone.0247462.s002.TIF]

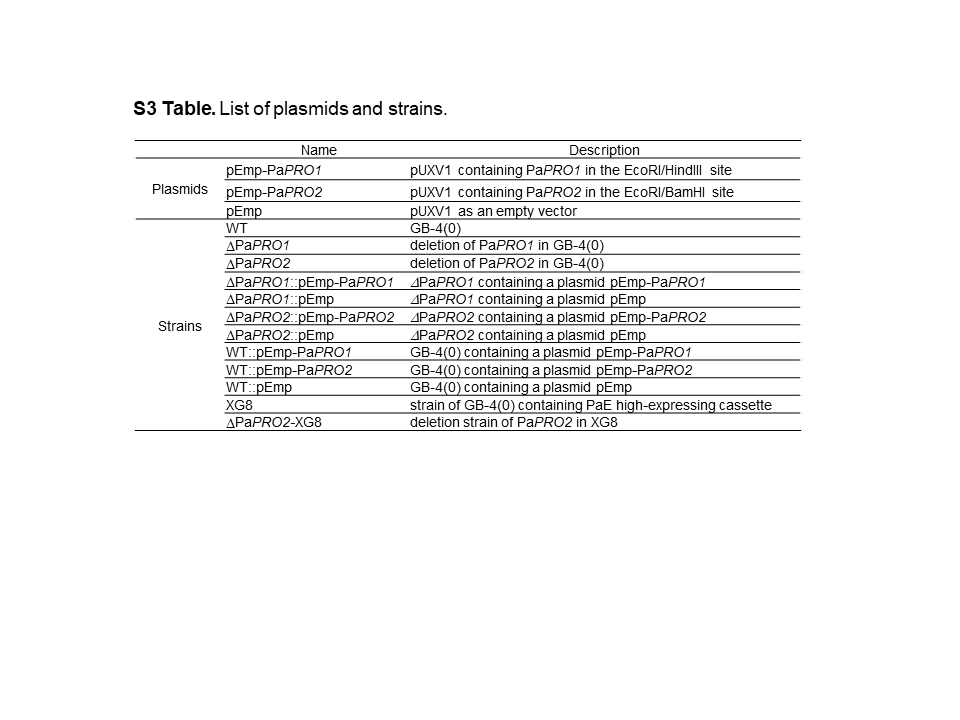

Supplement: S3 Table — (TIF) [file pone.0247462.s003.TIF]

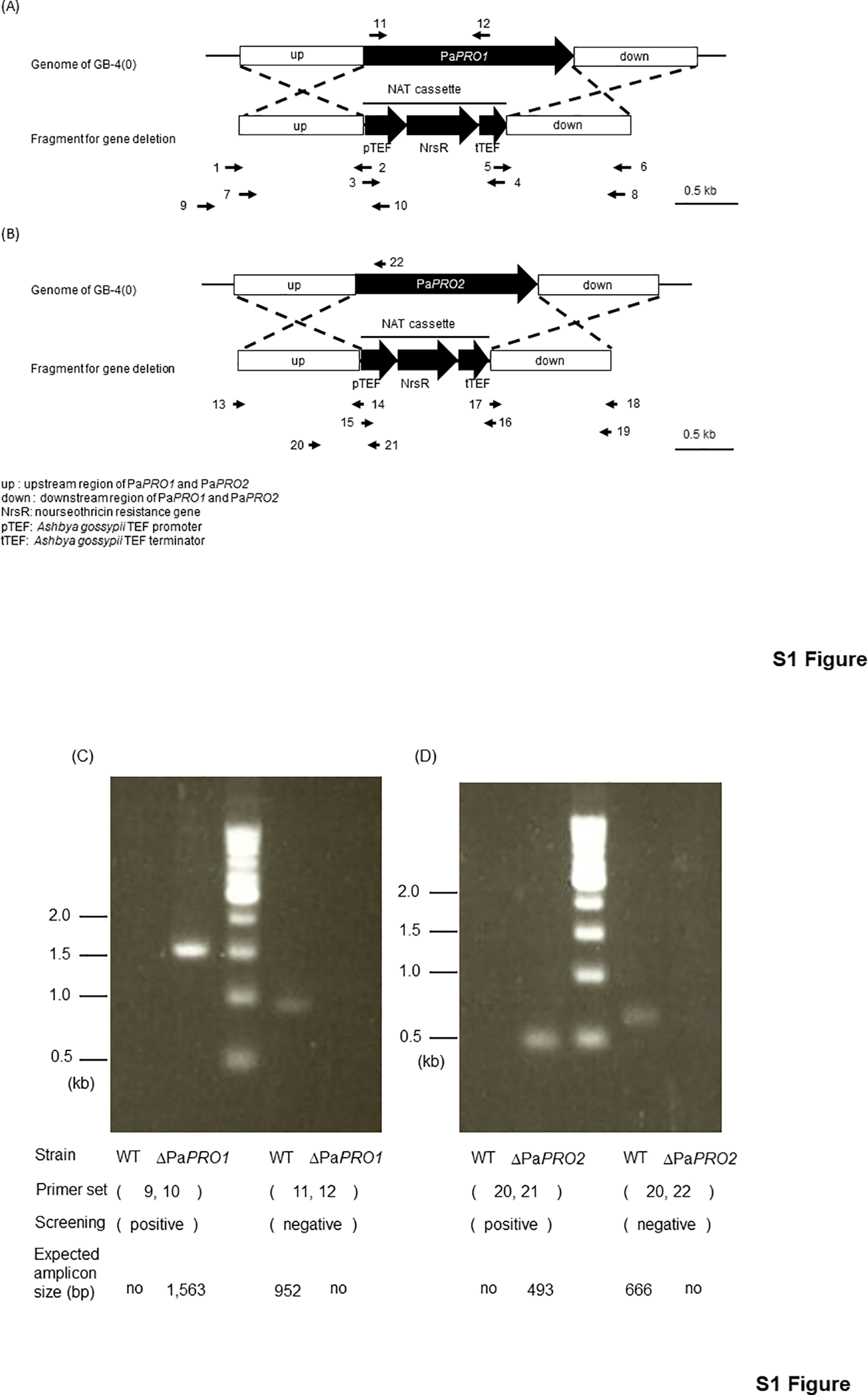

Supplement: S1 Fig — Disruptive fragments for PaPRO1 and PaPRO2 are shown in (A) and (B), respectively. Numbers and arrows indicate primers. Descriptions of primers and amplicons are provided in S1–S3 Tables. Deletion of the PaPRO1 (C) or PaPRO2 (D) gene was verified by PCR during positive and negative screening. (TIF) [file pone.0247462.s004.tif]

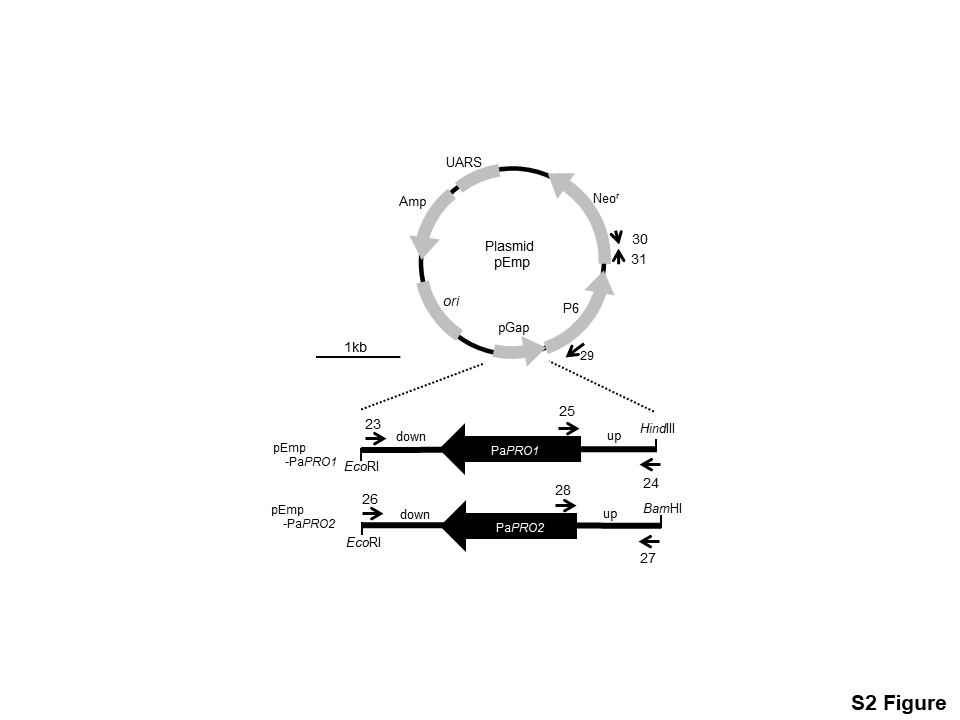

Supplement: S2 Fig — PaPRO1 or PaPRO2 containing flanking sequences was inserted into pEmp plasmid using restriction sites. (TIF) [file pone.0247462.s005.TIF]

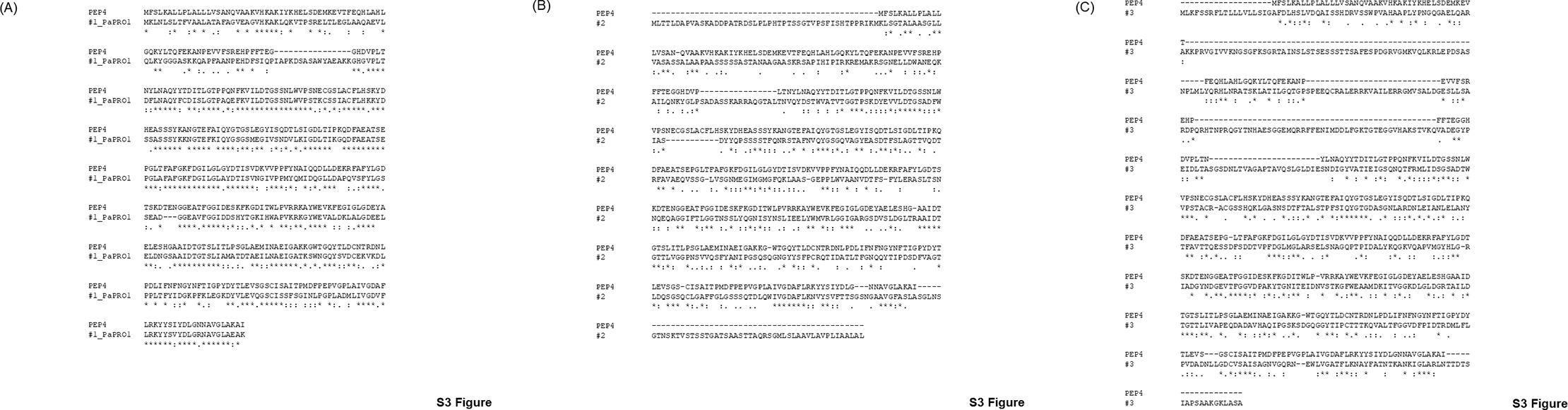

Supplement: S3 Fig — (A) candidate #1, (B) candidate #2, and (C) candidate #3. “*”, “:”, and “.”indicate identical residues, residues with strong similarities, and residues with weak similarities, respectively. (TIF) [file pone.0247462.s006.tif]

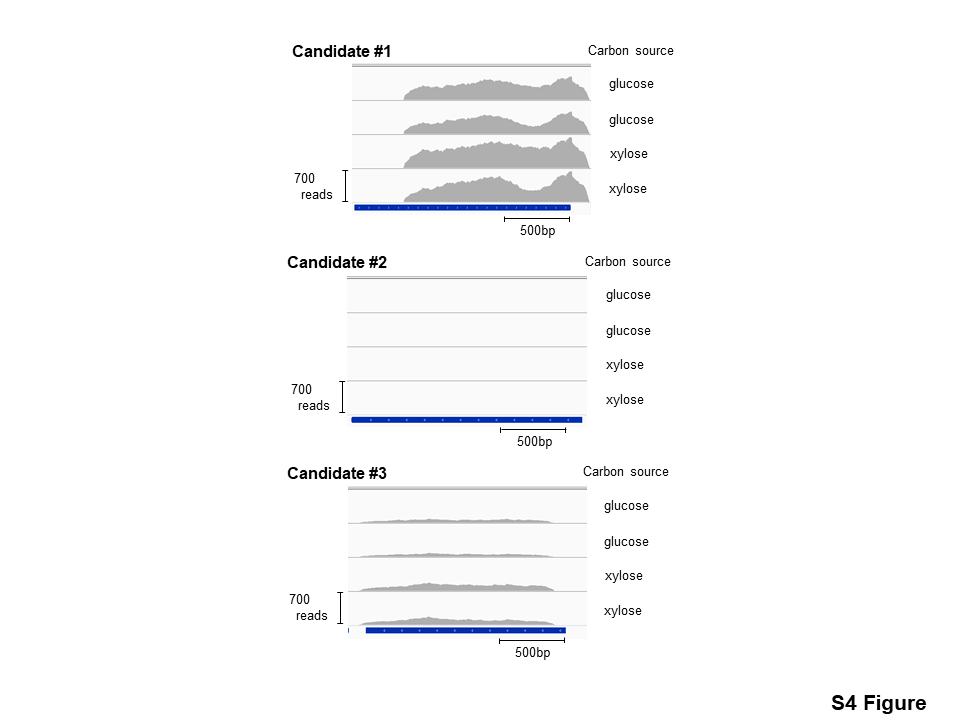

Supplement: S4 Fig — The expression frequency of the three orthologous gene was analyzed based on the read number obtained by sequence analysis of mRNA using MiSeq. Total RNA was isolated from cells using ISOGEN (Wako) according to the manufacturer’s instructions as follows. Cells were ground in liquid nitrogen to a fine powder, and approximately 3 g was mixed with 15 ml ISOGEN solution, followed by the addition of 3 ml chloroform. The mixture was left at room temperature for 3 min and centrifuged at 2,300 g for 20 min. The supernatant was transferred to a fresh tube and mixed with 7.5 ml isopropanol. The mixture was left at room temperature for 10 min and centrifuged at 2,300 g for 20 min. The pellet was dried and dissolved in 300 μl of RNase-free water. mRNA was purified from approximately 200 μg total RNA using an OligotexTM-dT30 mRNA Purification kit (Takara, Shiga, Japan) according to the manufacturer’s instructions. The library was prepared with TruSeq Stranded mRNA Library Prep (Illumina Inc., San Diego, CA, USA), and obtained the sequences with MiSeq (Illumina) according to the manufacturer’s instructions. Read number of each nucleotid was viewed with IGV [30]. (TIF) [file pone.0247462.s007.TIF]

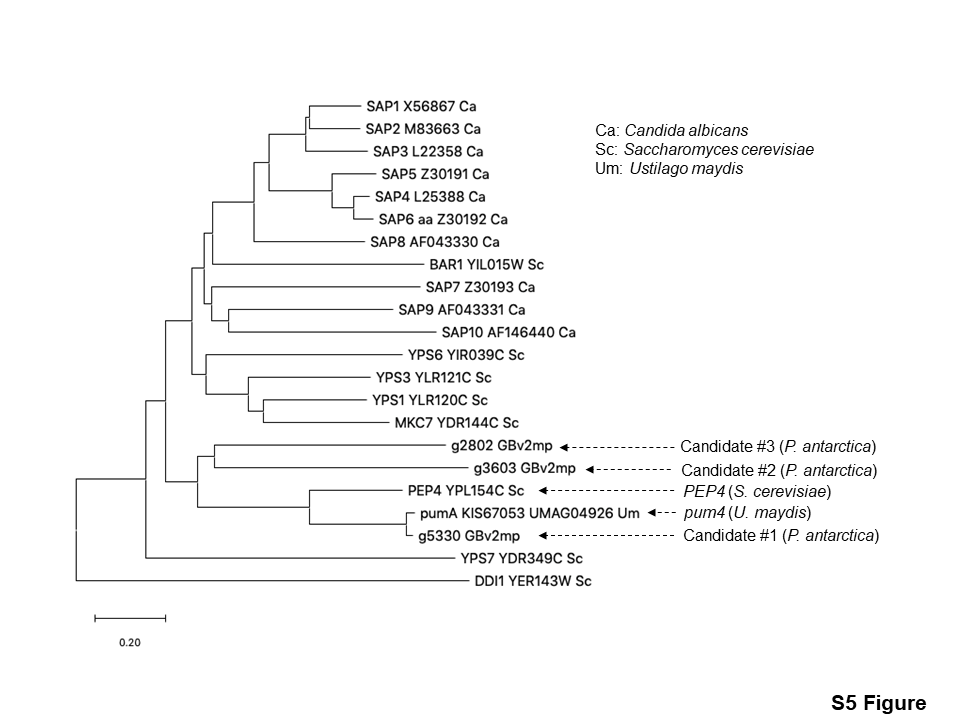

Supplement: S5 Fig — The evolutionary distance was calculated with Poisson correction method [32] and the tree was shown in scale. This analysis was performed in MEGA X [33, 34]. (TIF) [file pone.0247462.s008.TIF]

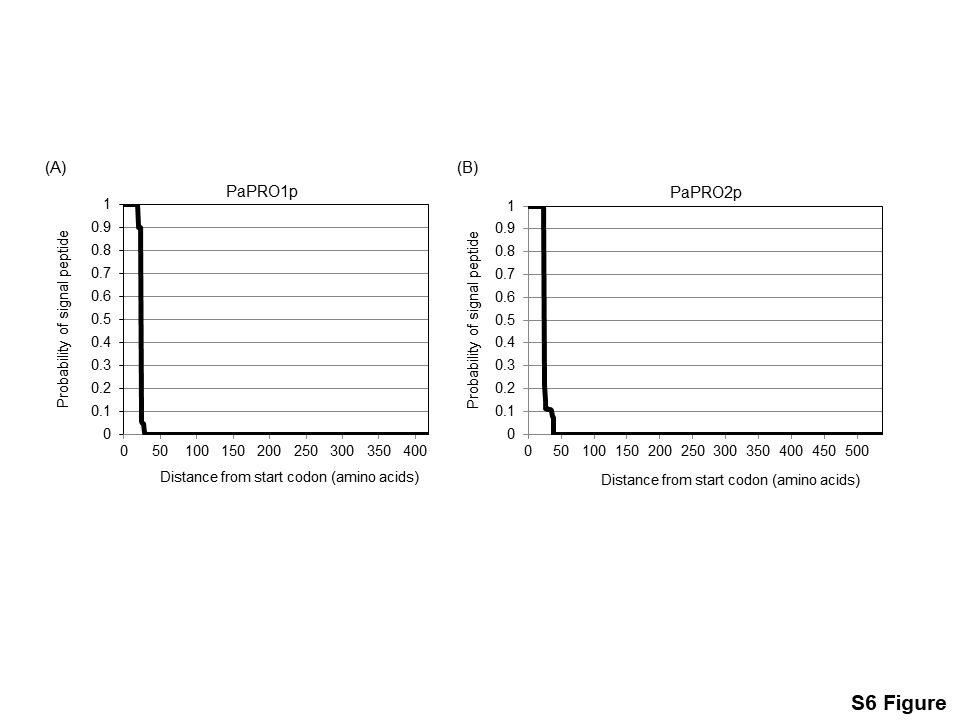

Supplement: S6 Fig — Proposed signal peptides of PaPRO1p and PaPRO2p according to Phobius are shown in (A) and (B), respectively. Horizontal axes show the distances of amino acid residues from the start codon; vertical axes show the probability of a signal peptide (maximum value = 1). (TIF) [file pone.0247462.s009.TIF]

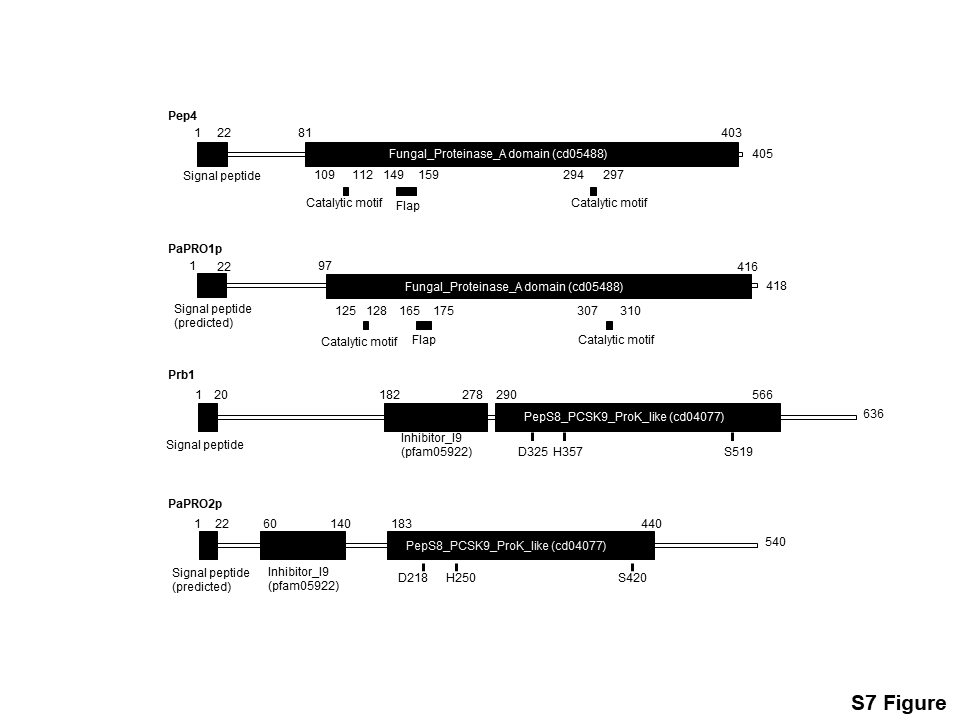

Supplement: S7 Fig — Conserved domains were predicted by BLASTP. Numbers indicate the positions of amino acid residues. (TIF) [file pone.0247462.s010.TIF]

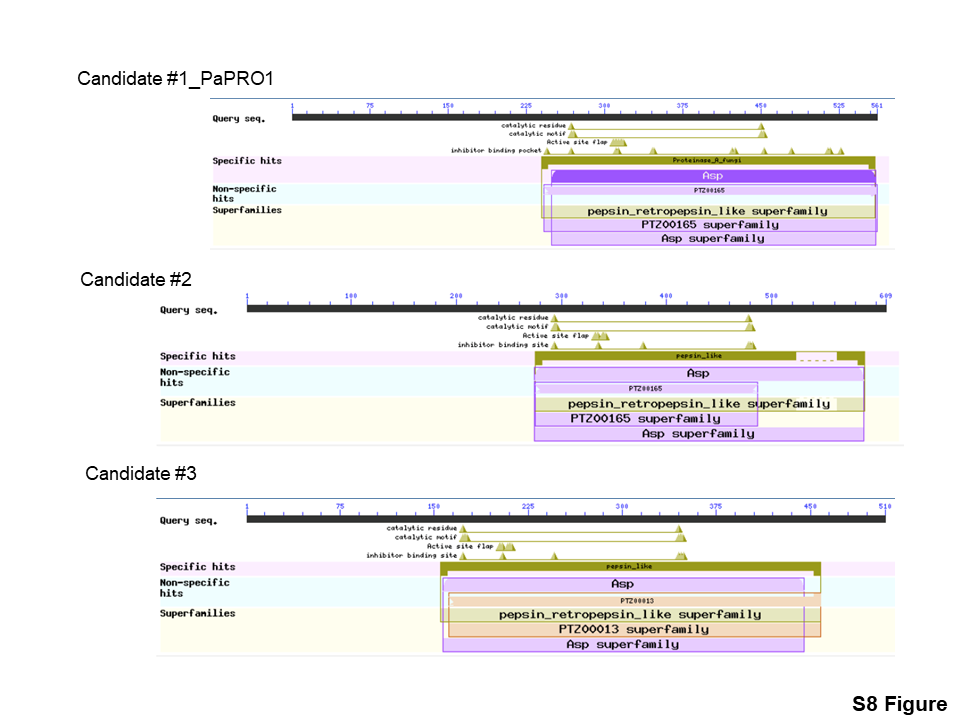

Supplement: S8 Fig — (TIF) [file pone.0247462.s011.TIF]

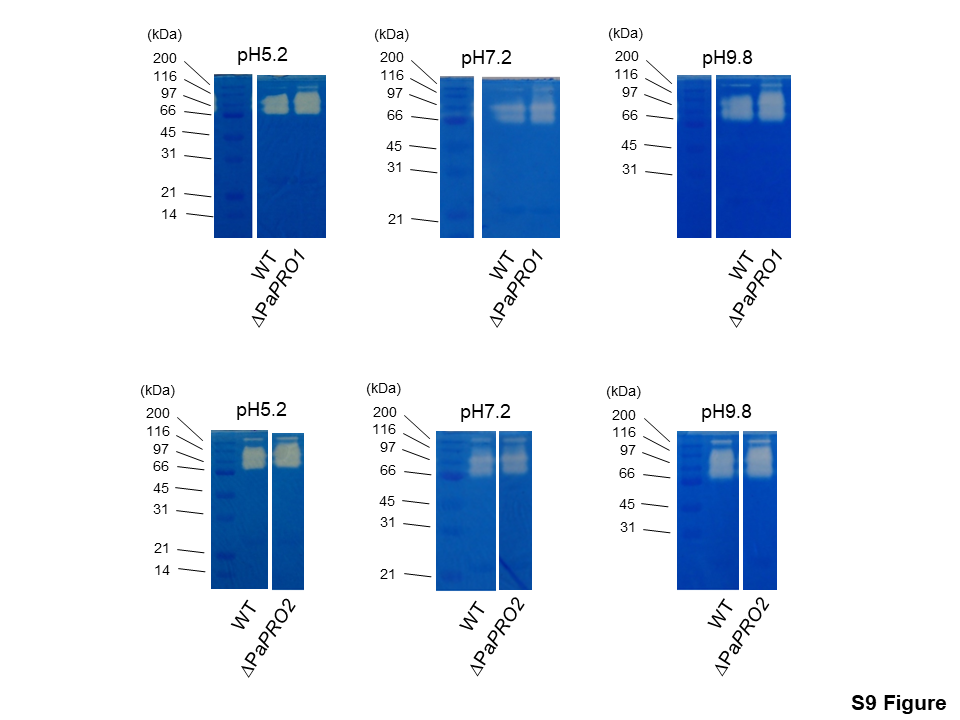

Supplement: S9 Fig — Culture supernatants of ΔPaPRO1, ΔPaPRO2, and wild-type GB-4(0) were centrifuged at 20, 000 × g for 5 min, and subjected to SDS-PAGE containing 0.1% gelatin without heat denaturation. After electrophoresis, to remove SDS [35], the polyacrylamide gel was incubated with 2.5% tritonX-100 at 25°C for 1h, washed with distilled water, and then incubated with buffer fluids, i.e, 0.1M sodium acetate (pH5.2), 0.1M Tris-HCl (pH7.2), or 0.1M Tris-HCl (pH9.8). The gel was stained with Symply Blue Safe Stain (Invitrogen, CA). Protease activity was visualized as white band caused by gelatin degradation. (TIF) [file pone.0247462.s012.TIF]

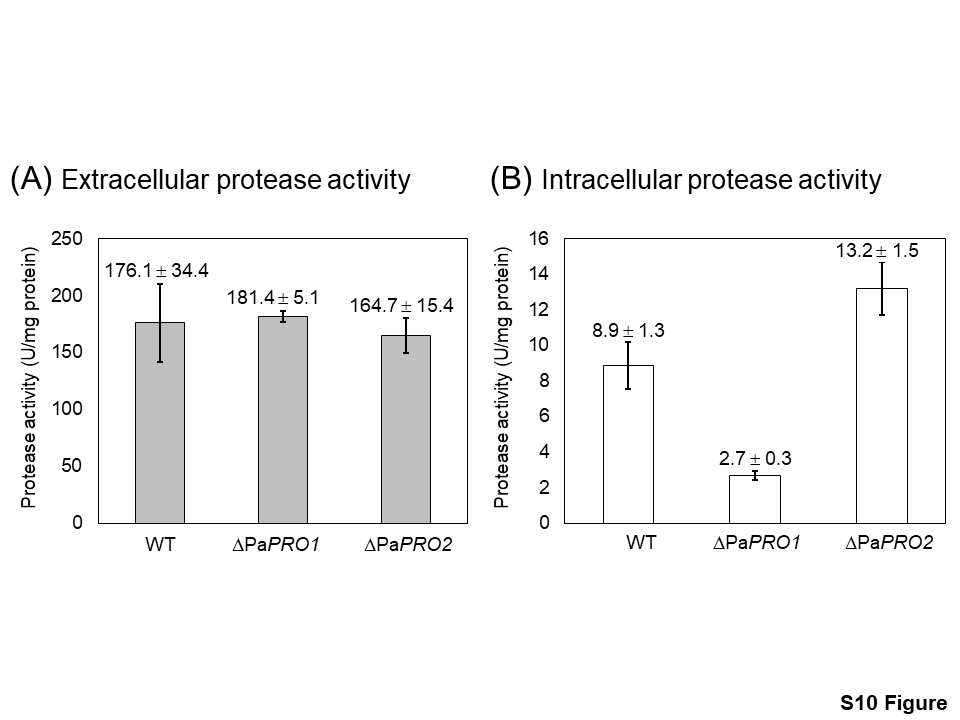

Supplement: S10 Fig — The activity of extra- (A) and intracellular (B) acid proteases was determined in the same manner as published studies [14, 36] with minor modifications. All strains were cultivated at 30°C for 5 days as with the flask cultivation in the Materials and methods section. The 1 mL of cultures after the cultivation were collected by a centrifuge at 14,000 g for 2 min and the supernatants were used as extracellular enzyme samples. The cell pellets were washed by 1 mL of 150 mM NaCl, resuspended in 1 mL of pure water. The resuspended cells (1 mL) were disrupted using the bead beater homogenizer (μT-12, TAITEC, Saitama, Japan) and zirconia beads (20 beads with 2 mm diameter and 3 beads with 3 mm diameter in 2 mL tube). After the disruption (3200 rpm for 20 sec and cooling on ice for 1 min, the cycle was repeated for 15 times), the disrupted cells were centrifuged at 22,000 g for 5 min at 4°C, then the supernatants were used as intracellular enzyme samples. The acid-denatured hemoglobin from bovine blood (Product No. H2625, Sigma Aldrich, St. Louis, MO, USA) was used as a substrate. The denaturation was performed by the incubation of hemoglobin in HCl (pH 1.8) at 35°C for 1 h, followed by pH adjustment at 3.2 by NaOH (Final concentration of hemoglobin was 20 g/L). The enzyme samples (400 μL) was mixed with 400 μL of acid-denatured hemoglobin (20 g/L, pH 3.2) and 400 μL of glycine-HCl buffer (100 mmol/L, pH 3.2) at 37°C. A portion (390 uL) of the mixture was taken at 0, 30, and 60 min, then, mixed with 700 μL of ice-cold trichloroacetic acid (TCA, 50 g/L) to stop the reaction. The TCA containing samples were incubated for 20 min at room temperature to progress the denaturation. Obtained samples were centrifuged at 22,000 g for 5 min at 4°C to remove denatured hemoglobin and cell-derived proteins, and these supernatants (500 μL) were mixed with 500 μL of 1 M NaOH. Finally, tyrosine-containing peptide in soluble fraction was determined using Folin & Ciocalteu’s phenol reagent ( [file pone.0247462.s013.TIF]

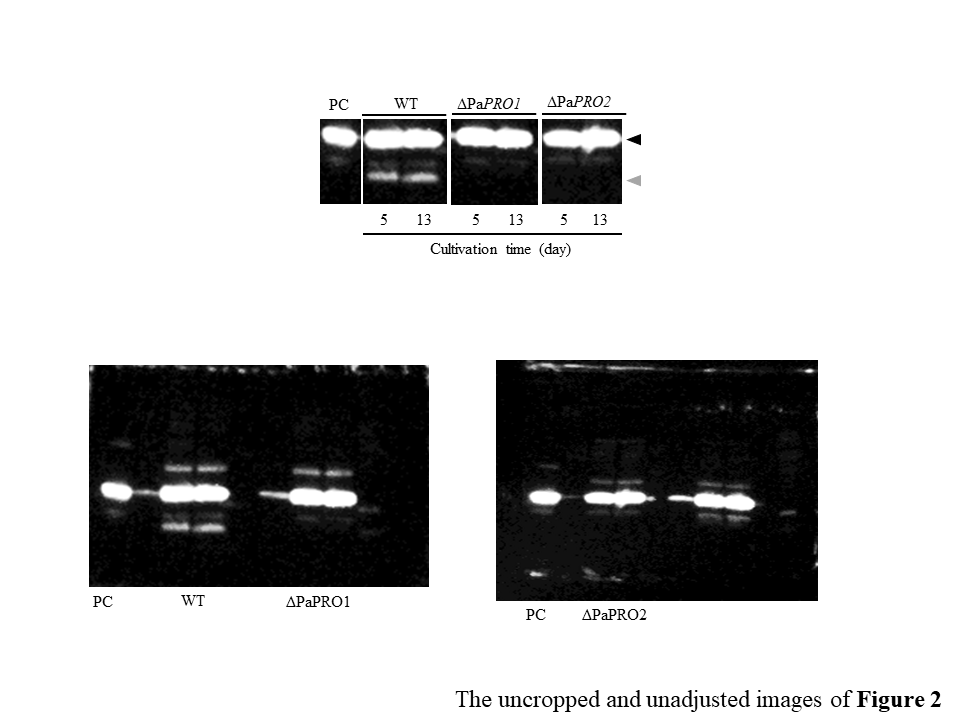

Supplement: S1 Raw image — (TIF) [file pone.0247462.s014.TIF]

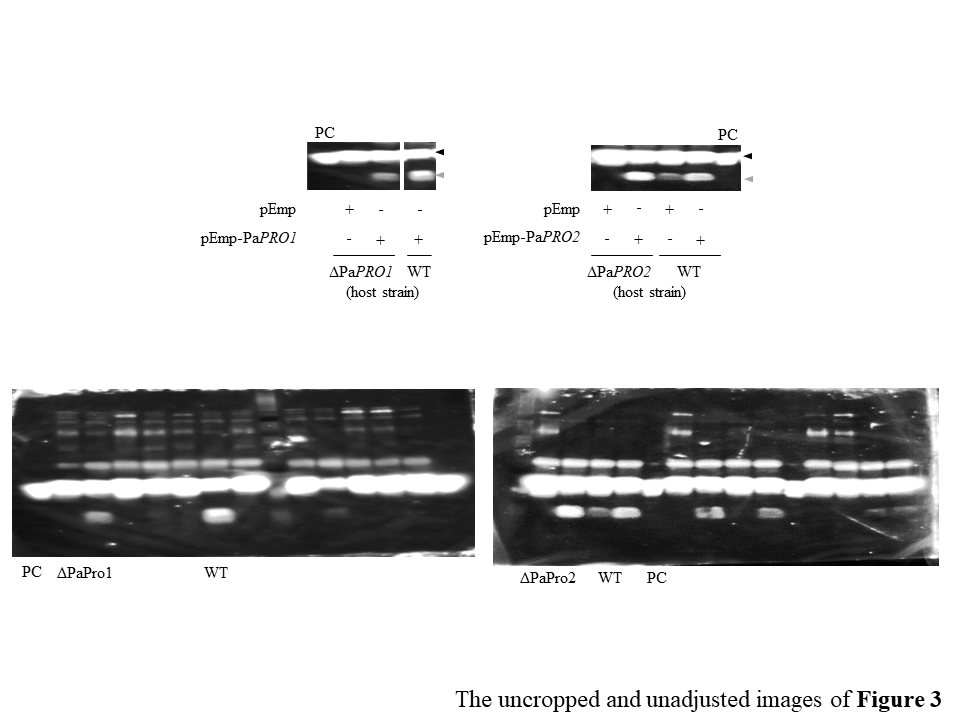

Supplement: S2 Raw image — (TIF) [file pone.0247462.s015.TIF]

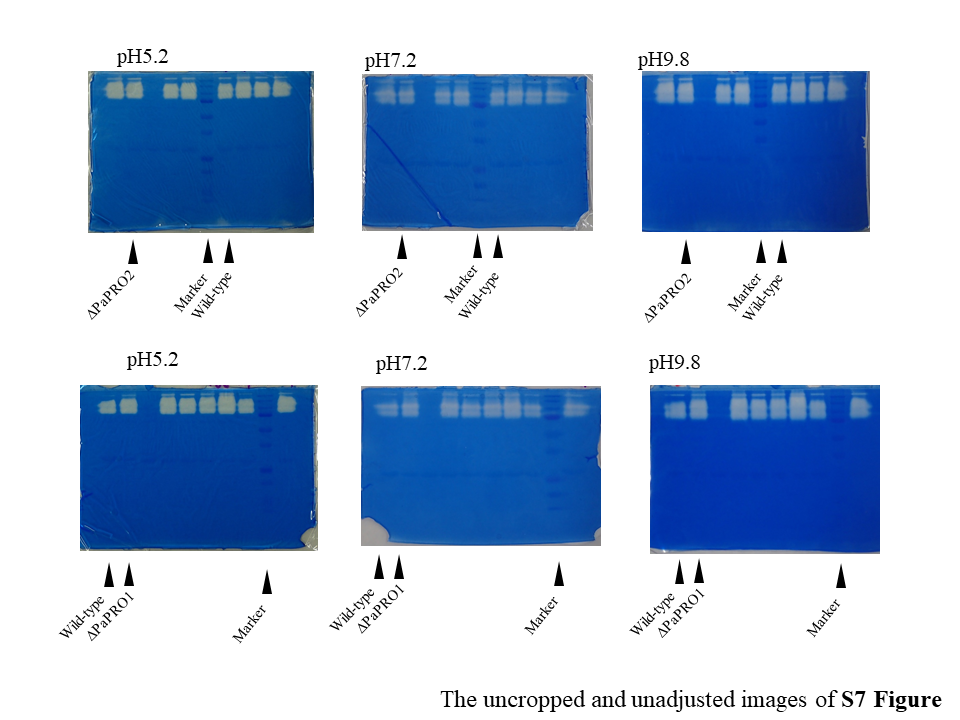

Supplement: S3 Raw image — (TIF) [file pone.0247462.s016.TIF]
